# Further identification of a 140bp sequence from amid intron 9 of human *FMR1* gene as a new exon

**DOI:** 10.1186/s12863-020-00870-2

**Published:** 2020-06-18

**Authors:** Wen-jing Yang, Ai-zhen Yan, Yong-jun Xu, Xiao-yan Guo, Xian-guo Fu, Dan Li, Juan Liao, Duo Zhang, Feng-hua Lan

**Affiliations:** 1 Department of Clinical Genetics and Experimental Medicine, 900th Hospital of the Joint Logistics Force, Xiamen University School of Medicine, 156 Xi’erhuanbei Road, Fuzhou City, Fujian Province 350025 People’s Republic of China; 2 Present addresses: Department of Laboratory Medicine, Zhongshan Hospital, Fudan University, Shanghai, 200032 China; 3 Present addresses: Department of Laboratory Medicine, Fuzhou No. 2 Hospital Affiliated Xiamen University, Fuzhou, Fujian 350007 People’s Republic of China; 4 Present addresses: Department of Laboratory Medicine, Ningde Municipal Hospital, Fujian Medical University, Ningde City, 352100 Fujian Province China; 5 Present addresses: Department of Laboratory Medicine, Fujian University of Traditional Chinese Medicine Affiliated People’s Hospital, Fuzhou, 350001 Fujian China

**Keywords:** *FMR1*, FMRP, FXS, Alternative splicing, Alternative exon

## Abstract

**Background:**

The disease gene of fragile X syndrome, *FMR1* gene, encodes fragile X mental retardation protein (FMRP). The alternative splicing (AS) of *FMR1* can affect the structure and function of FMRP. However, the biological functions of alternatively spliced isoforms remain elusive. In a previous study, we identified a new 140bp exon from the intron 9 of human *FMR1* gene. In this study, we further examined the biological functions of this new exon and its underlying signaling pathways.

**Results:**

qRT-PCR results showed that this novel exon is commonly expressed in the peripheral blood of normal individuals. Comparative genomics showed that sequences paralogous to the 140 bp sequence only exist in the genomes of primates. To explore the biological functions of the new transcript, we constructed recombinant eukaryotic expression vectors and lentiviral overexpression vectors. Results showed that the spliced transcript encoded a truncated protein which was expressed mainly in the cell nucleus. Additionally, several genes, including the *BEX1* gene involved in mGluR-LTP or mGluR-LTD signaling pathways were significantly influenced when the truncated FMRP was overexpressed.

**Conclusions:**

our work identified a new exon from amid intron 9 of human *FMR1* gene with wide expression in normal healthy individuals, which emphasizes the notion that the AS of *FMR1* gene is complex and may in a large part account for the multiple functions of FMRP.

## Background

Fragile X syndrome (FXS) is a common form of inherited intellectual disability, affecting 1/7000 females and 1/4000 males. Apart from mild to severe intellectual disability, individuals with FXS often exhibit autism-like behavior, including social anxiety, attention deficit, mood disturbance, and sleep disorders. FXS patients also present other prominent physical symptoms, encompassing a long, narrow face with large protruding ears, flat feet, eye-gaze avoidance, and macroorchism. Pathogenically, when the trinucleotide CGG repeat in the 5′ untranslated region of the fragile X mental retardation 1 (*FMR1*) gene expands to more than 200, DNA methylation and transcriptional silencing of *FMR1* will be occur, leading to the loss of *FMR1* gene product, that is, fragile X mental retardation protein (FMRP) [1]

In mammals, full-length *FMR1* gene sequence encodes a 71 kDa FMRP. FMRP has three motifs, including two K homology domains (KH1 and KH2) encoded by exons 7–9 and exons 9–13, respectively, and the arginine-glycine-glycine (RGG) box encoded by exons 15–16, that mediates RNA or protein interaction. FMRP also contains a nuclear localization signal (NLS) and a nuclear export signal (NES). Additionally, numerous studies showed that FMRP has agenet domains at N terminus to combine with methylated H3K9 chromatin [2] FMRP is also a selective mRNA-binding protein that regulates RNA transcription, splicing, and cell apoptosis [3] It is estimated that FMRP can bind more than 5% mRNA in cells. Yeast three-hybrid assay and microarray have detected as many as 400 potential mRNAs related to FMRP [4] FMRP is widely expressed and especially abundant in brain, playing a critical role in synaptic plasticity and neurological signaling pathways as a translational repressor. Among them, metabotropic glutamate receptor–long-term depression (mGluR-LTD) is one of the most common pathways that can regulate the α-amino-3-hydroxy-5-methyl-4-isoxazolepropionic acid receptor (AMPAR) internalization and local protein synthesis to prevent deficits in synaptic plasticity [5] In addition, mGluR-long-term potentiation (mGluR-LTP), phosphorylation of FMRP, gamma-aminobutyric acid (GABA), and dopamine receptors (DA) signaling pathways are all required for the formation of normal neurological function [6]

Mature transcripts from *FMR1* gene have multiple alternatively spliced isoforms in different organs. The most common ways of alternative splicing (AS) are the inclusion or exclusion of exons 12 and 14, and the selection of splice acceptor sites on exons 15 and 17 [7] *FMR1* has been reported to produce more than 20 FMRP isoforms with various structures and functions. Different FMRP isoforms may be involved in various signal transduction pathways, implying their significant biological functions. With the incidence of 50 to 75% of in central nervous system, alternatively spliced genes have important influence on synaptic plasticity [8] ion channel activity [9] the genesis of dendritic spines [10] and the release of neurotransmitters [11] *FMR1* is an example of key gene that produces various spliced transcripts in human brain. However, the exact number of alternatively spliced isoforms in various tissues and cells and their specific biological functions are still poorly understood.

Previously, we and others reported a novel 140 bp exon when detecting alternatively spliced transcripts from *FMR1* gene [12, 13] This 140 bp sequence comes from intron 9 of human *FMR1* gene and introduces a premature stop codon in the resulting mRNAs. Bioinformatics analysis shows that the novel sequence has canonical splicing signals, implying that its potential as an alternative exon. Furthermore, qRT-PCR showed the exon can be detected in mature mRNA molecules of the peripheral blood from non-FXS individuals. Using eukaryotic expression vector and lentiviral vector for truncated FMRP analysis, we determined that the inclusion of the novel 140 bp sequence leads to a truncated protein with altered subcellular distribution. RNA microarray analysis on cells overexpressing the truncated FMRP revealed a group of differentially expressed genes that might contribute to the FMRP signaling pathways. Our work emphasizes the notion that the AS of *FMR1* gene is considerably more complex than what we have realized, and such complexity may in a large part account for the multiple functions of FMRP.

## Results

### Comparative genomics shows that sequences paralogous to the 140 bp sequence only exist in the primate genomes

**Fig. 1:**
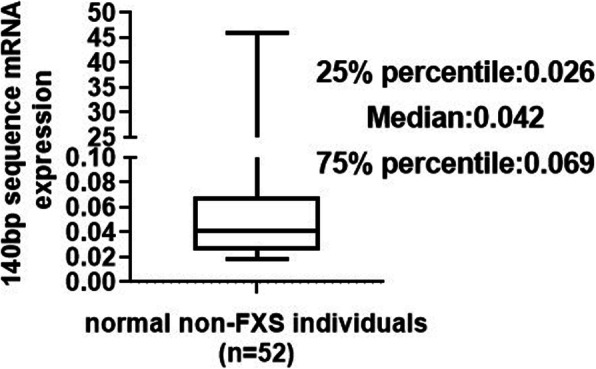
The new exon of 140 bp is commonly expressed and distributed in the peripheral blood of normal non-FXS individuals. qRT-PCR results of mRNA expression level of the 140 bp sequence in peripheral blood cells of 52 non-FXS controls

To determine the relative abundance of FMR1 gene expression in the peripheral blood of normal non-FXS individuals, real-time reverse transcriptase PCR (qRT-PCR) was carried out using specific primers (Supplementary Tab. S1) aiming at the 140 bp sequence. Box plot revealed that the first quartile (25% percentile) of 140 bp mRNA expression was 0.026, the second quartile (median) was 0.042, and the third quartile (75% percentile) was 0.069. Among them, the minimum value of 140 bp mRNA expression was 0.0187, and the maximum value was 45.960 (Fig. 1). These results indicated that FMR1 mRNA containing the 140 bp sequence exists ubiquitously in the peripheral blood of normal healthy individuals.

We further searched the genomes of other species for sequences similar to this new spliced variant. Genomes of *FMR1* gene from diverse species were obtained and submitted to DANMAN software. The results showed that primates, such as *Homo sapiens* (GenBank: NC_000023), *Macaca mulatta* (GenBank: NC_012614), *Pan troglodytes* (GenBank: NC_006491), and *Pongo abelii* (GenBank: NC_007878), all have paralogues of the 140 bp sequence, although they are distributed in different introns of *FMR1* gene, for example, in intron 9 of *Homo sapiens* and *Macaca mulatta* and in intron 10 of *Pan troglodytes* and *Pongo abelii* (Supplementary Fig. S1). However, the 140 bp sequence is absent in non-primates, implying that the 140 bp sequence might be associated with the development and evolution of intelligence.

### FMR1 mRNA containing the 140 bp sequence can be translated into a truncated FMRP with altered cellular localization

**Fig. 2:**
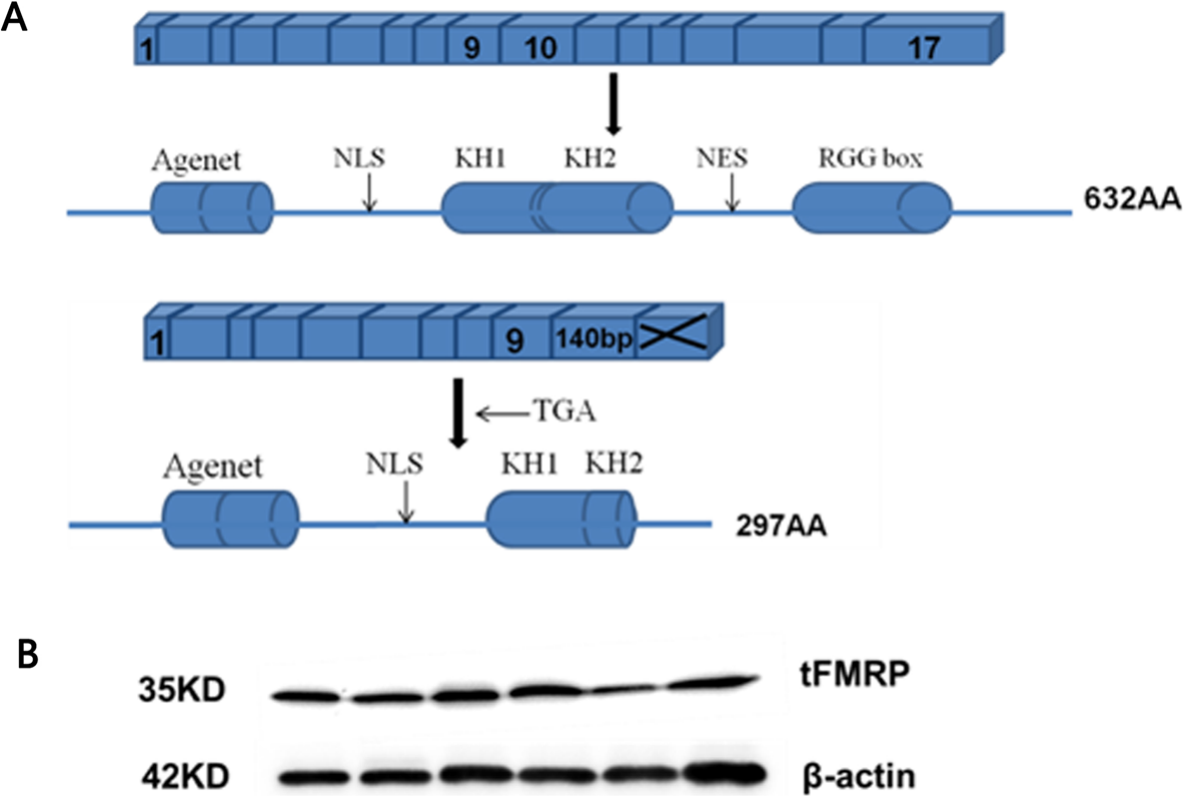
The structure and expression of truncated FMRP protein in healthy individuals. **a** FMRP structure with coding exons (squares) and functional domains (columns). **a**: FMR1 gene containing full-length sequence encodes 632 amino acids; **b**: coding fragment containing exons 1–9 and the 140 bp sequence. **b** Western blots showing the expression of the new alternatively spliced FMR1 transcript in peripheral blood cells of 6 healthy individuals. tFMRP: truncated FMRP

The splicing of the novel exon with exon 9 at the 5′ end and exon 10 at the 3′ end results in *FMR1* mRNAs that can be translated into a 34 kDa truncated FMRP containing 297 amino acid residuals, while lacking a large part of the carboxy-terminal domains, such as NES, the second KH domain, and RGG box (Fig. 2a). To identify the potential truncated protein and detect the expression of this newly alternative *FMR1* transcript, we extracted the total proteins from peripheral blood cells of six normal non-FXS individuals and performed Western blot using a monoclonal antibody against the N-terminus of FMRP. The results showed that a protein band with a molecular weight of approximate 35 kDa, which is almost half the length of full-length FMRP could be seen in all six normal individuals (Fig. 2b).

**Fig. 3:**
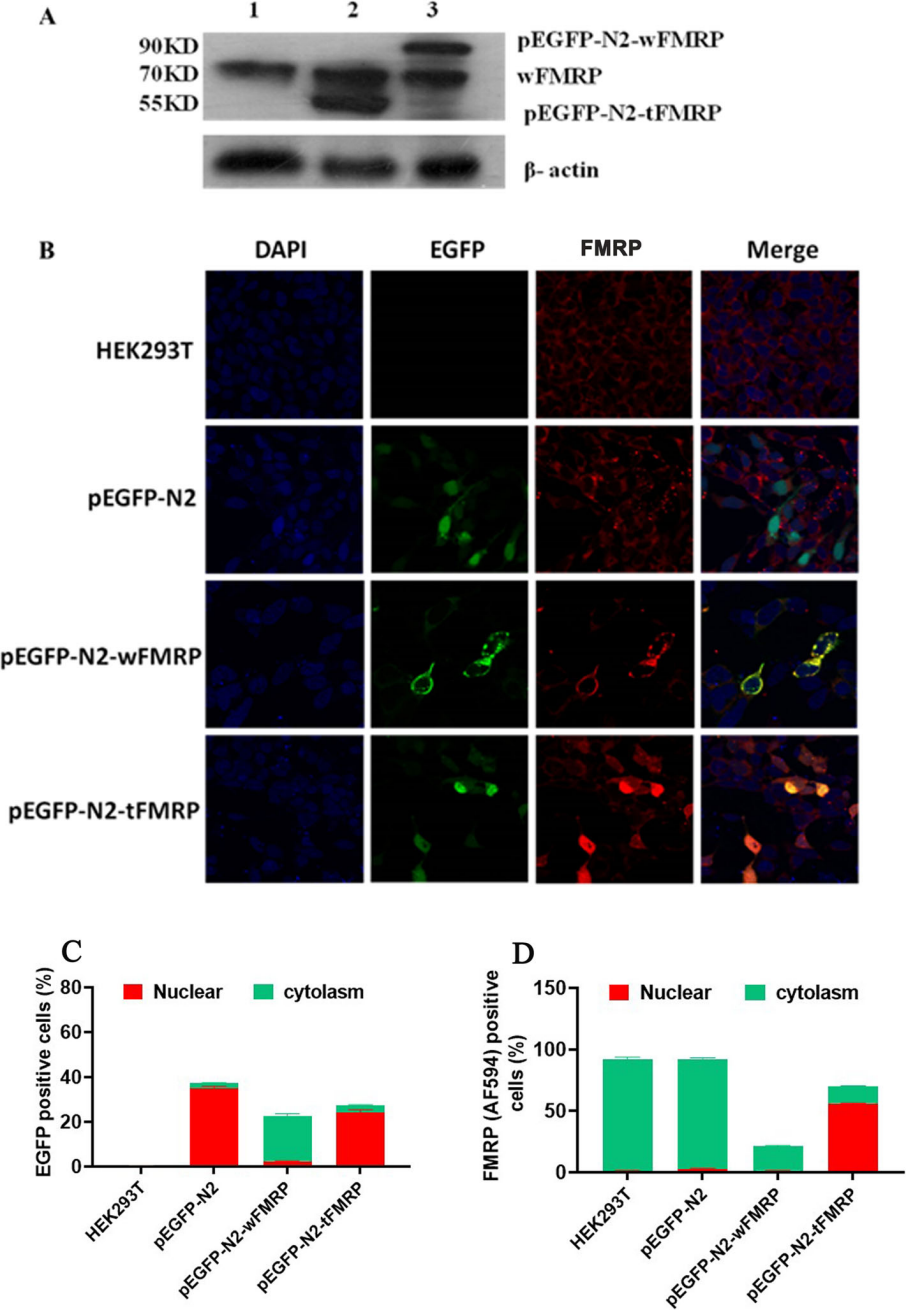
The new spliced transcript containing the 140 bp sequence is translated into a truncated FMRP protein with altered subcellular localization. **a** Western blots showing the expression of the wild type FMRP or the truncated FMRP in HEK293T cells. 1: untransfected HEK293T cells; 2: HEK293T cells transfected by pEGFP-N2-tFMR1; 3: HEK293T cells transfected by pEGFP-N2-fFMR1. **b** Immunofluorescence showing the localization of the wild type FMRP or the truncated FMRP in HEK293T cells. HEK293T: untransfected HEK293T cells; pEGFP-N2: HEK293T cells transfected by the empty vector; pEGFP-N2-tFMR1: HEK293T cells transfected by pEGFP-N2-tFMR1; pEGFP-N2-fFMR1: HEK293T cells transfected by pEGFP-N2-fFMR1; DAPI: the solution suitable for nuclear staining; EGFP: vectors encoding the GFP-tagged protein; Alexa Flour 594: a FMRP antibody fluorescently labeled red. **c** Quantitative representation of EGFP positive cells. **d** Quantitative representation of FMRP positive cells

To explore the subcellular localization of the end product from the new alternatively spliced *FMR1* transcript, we constructed recombinant eukaryotic expression vectors with full-length coding sequence (pEGFP-N2-fFMR1, full-length isoform) or coding fragment containing exons 1–9 together with the 140 bp sequence (pEGFP-N2-tFMR1, truncated isoform), respectively. Western blot showed that HEK293T cells transfected by void plasmid, pEGFP-N2-fFMR1, and pEGFP-N2-tFMR1 all expressed a 71 kDa endogenous FMRP. HEK293T cells transfected by pEGFP-N2-fFMR1 and pEGFP-N2-tFMR1 expressed FMRP or truncated FMRP fused with EGFP, with molecular weights of 90 and 55 kDa, respectively (Fig. 3a). Immunofluorescence and quantitative representation of EGFP and FMRP positive cells revealed that the full-length FMRP was expressed mainly in the cytoplasm, whereas the truncated protein mainly existed in the cell nucleus, demonstrating an altered subcellular localization (Fig. 3b-d).

### FXS-related signal pathways are significantly influenced when the truncated FMRP is overexpressed

**Fig. 4:**
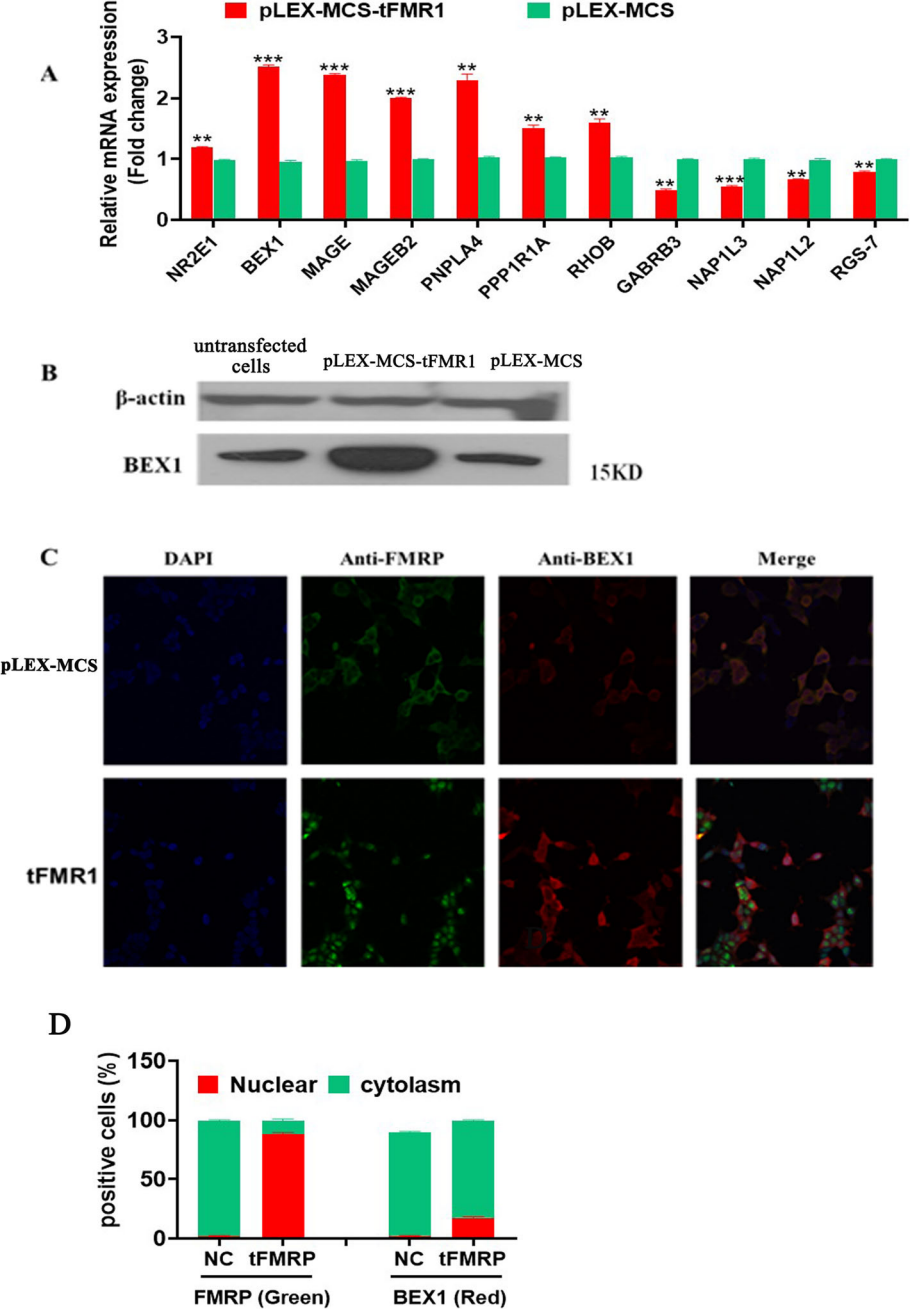
Overexpression of the truncated FMRP protein altered FXS-related signaling pathways. **a** Partial qRT-PCR results of differentially expressed genes. Red: HEK293T cells transfected by pLEX-MCS-tFMR1; Green: HEK293T cells transfected by pLEX-MCS. **b** Western blots showing the overexpression of *BEX1* protein in HEK293T cells transfected by pLEX-MCS-tFMR1. 1: untransfected HEK293T cells; 2: HEK293T cells transfected by pLEX-MCS-tFMR1; 3: HEK293T cells transfected by pLEX-MCS. **c** Immunofluorescence showing the localization of *BEX1* protein in HEK293T cells. HEK293T: untransfected HEK293T cells; pLEX-MCS-tFMR1: HEK293T cells transfected by pLEX-MCS-tFMR1. DAPI: the solution suitable for nuclear staining; Anti-FMRP: the primary antibodies of FMRP; Anti-BEX1: the primary antibodies of BEX1. **d** Quantitative representation of FMRP and BEX1 positive cells. **p < 0.01; ***p < 0.001

RNA microarray analysis revealed 545 genes with altered expression (the top 20 up-regulated and down-regulated differentially expression genes are shown in Supplementary Tab. S2 and Tab. S3, respectively), among which *BEX1* (brain expressed X-linked 1) is the most significantly up-regulated gene, and *GABRB3* (gamma-aminobutyric acid type A receptor beta3 subunit) is the most significantly down-regulated gene (Supplementary Tab. S2 and Tab. S3, respectively). To verify the microarray results, we selected 11 genes for qPCR, which demonstrated that 9 genes (*BEX1*, *MAGE*, *MAGEB2*, *PNPLA4*, *PPP1R1A, GABRB3, NAP1L3, NAP1L2,* and *RGS-7*) whose expression levels were altered to an extent similar to that shown in mRNA microarray analysis (Fig. 4a). Western blot showed that *BEX1* gene was overexpressed in HEK293T cells stably transfected by pLEX-MCS-tFMR1 (Fig. 4b). Immunofluorescence and quantitative representation of FMRP and BEX1 positive cells revealed that *BEX1* gene was expressed both in the cytoplasm and cell nucleus, but it was mainly located in the cytoplasm of HEK293T cells, co-localizing with the endogenous FMRP (Fig. 4c&d).

We performed an integrated bioinformatics analysis on 545 differentially expressed genes (DEGs). Ontological classification revealed that the DEGs were mainly enriched in three categories, including biological process (BP), cellular component (CC), and molecular function (MF). In the BP group, 28% proteins are concerned with metabolic process and 19% with cellular process, whereas the DEGs in the CC group comprise of cell part (37%), organelle (25%), and membrane (16%). For the MF group, the DEGs are associated with binding (45%) and catalytic activity (31%) (Supplementary Fig. S2C). KEGG analysis demonstrated that the DEGs are mainly involved in four significant signaling pathways, i.e. cancer-related pathways, PI3K-Akt, influenza A, and neuroactive ligand-receptor interaction signaling pathways.

## Discussion

### Alternative splicing of FMR1 gene

Almost 95% human genes would experience certain level of alternative splicing (AS) and contribute to proteome complexity [14] AS can also produce mRNAs that differ in their untranslated regions or coding sequence. The mechanisms of AS mainly include exon skipping, intron retention, the use of alternative splice sites, and the choice of mutually exclusive exons. The different spliced isoforms might influence mRNA localization, stability and translation. Moreover, several splicing mRNA variants could alter the reading frame and generate various protein isoforms with diverse localizations and functions. The most common site for AS is in the neural tissue, where various spliced transcripts may function as modulators to synaptic functions. Since the cloning of *FMR1* gene as the disease gene of FXS in 1991, a large number of *FMR1* mRNAs and FMRP isoforms derived from AS have been detected in mouse and human [15] The distribution of different FMRP isoforms with specific cellular roles and in various tissues is also relatively well understood. It is reported that a high expression level of different *FMR1* mRNAs was observed in the brain, testis, placenta, and lymphocytes, whereas a low level of expression was noted in other organs [15] At least 4 predicted FMRP isoforms were identified in mouse brain, which demonstrates that the dissimilar isoforms of FMRP occur together in the same cell type or separately in distinct cell types [16] At present, more than 24 predicted mature transcripts have been reported, and they mainly involve the inclusion or exclusion of exons 12 and 14, and the selection of splice acceptor sites on exons 15 and 17 [17] To our knowledge, our work represents a further study identifying a new exon from intron 9 of human *FMR1* gene.

### Subcellular localization of FMRP

The longest isoform of FMRP (isoform 1) is predominantly cytoplasmic and mainly functions as mRNA-binding protein that can directly or indirectly interact with other proteins, regulating the stability of mRNA and maintaining the balance of shuttling between the cytoplasm and nucleus. The nuclear localization signal (NLS) and nuclear export signal (NES) of FMRP are also associated with the cytoplasmic localization of FMRP. A patient with a novel R138Q mutation in the NLS exhibits developmental delay [18] The mechanism of this mutation is unclear, but it may lead to the different distribution of proteins in cytoplasm and nucleus and may indicate the importance of the domain. In addition, exon 14 of *FMR1* gene encodes a cytoplasmic retention domain. Exclusion of exon 14 alters the downstream reading frame, generates two different C-terminal regions, and finally results in nuclear localization [7] Furthermore, FMRP C-terminal is one of the determinant factors of nuclear localization and is the key domain that mediates the kinesin and dendrite transmission [19] FMRP homologous proteins, including FXR1 and FXR2, also shuttle between the nucleus and cytoplasm by producing multiple isoforms with different C-terminal [20] Correspondingly, FMRP N-terminal is highly conserved in species [21] Banerjee and his colleagues studied the functional difference among long and short isoforms of FMRP of *D. melanogaster*, showing that the short isoforms, without the C-terminal region, can easily cause short-term or long-term learning and memory disorders [22]

FMRP can bind key protein cytoplasmic FMRP-interacting protein (CYFIP1), a downstream effector of Rac1 in the cytoplasm, remodeling the cytoskeleton and involving the formation of the translational initiation complex [23] However, previous studies presented that FMRP binds its mRNA targets in the nucleus and facilitates the cargo of nuclear proteins, and the export of FMRP from the nucleus depends on mRNA synthesis. Kim and his colleagues knocked down the mRNA exporter Tap/NXF1, resulting in the increased of FMRP levels in nucleus [24] It also has been proved that FMRP can combine proteins in nucleus, such as NUFIP1 and 82-FIP (FMR1 interacting protein 1), RISC (RNA-induced silencing complex), AGO2, and Dicer (argonaute 2), and eIF5 (eukaryotic translation initiation factor 5), which are pivotal molecules that mediate translational repression by inhibiting the initiation of translation and causing polyribosomes stalling [25, 26] Moreover, the combination process of FMRP and ribosomes being an important mechanism of translational regulation occurs in the nucleus. FMRP has been regarded as chromatin-associated protein. This protein can coimmunoprecipitate with chromatin as confirmed by Chip sequencing, and by interacting with nucleolin, affecting the transcription of rRNA and the biosynthesis of ribosomes [27]

Given the lack of nuclear export signal, our newly identified FMRP isoform with a short C-terminal retains in the nucleus. We speculate that the increase in FMRP alternatively spliced isoforms in the nucleus could disrupt the balance of shuttling between the nucleus and cytoplasm and affect the translational repression of FMRP.

### New interactors of FMRP in the FXS-related signaling pathways

With the development of several high-throughput approaches, such as microarray analysis, HITS-high-throughput sequencing of RNA isolated by crosslinking immunoprecipitation (CLIP) and PAR-photoactivatable ribonucleoside-enhanced CLI P (PAR-CLIP), research revealed that FMRP can interact with about 5% mRNA targets in the brain [4] FMRP is a translational repressor involved in the regulation of synaptic functions via the activation of NMDA receptors, AMPA receptors and GABA receptors, which contribute to the formation of long-term depression (LTD) and long-term potentiation (LTP), according to mGluR theory [28] It is widely believed that the regulation of neurological function mainly depends on the mGluR-LTD pathway, which mediates the synaptic plasticity and hinges on the local protein synthesis of dendrites. The mGluR theory of FXS emphasizes that FMRP is downstream of mGluRs and upstream of local protein synthesis. FMRP has been suggested to repress the translation of dendritically localized mRNAs. With the activation of mGluR, FMRP repression would allow the synthesis of local protein in response to synaptic stimulation, resulting in the AMPAR internalization and LTD [28] For patients with FXS, the absence of FMRP could constructively increase protein synthesis, leading to the over activation of AMPAR internalization and LTD exaggeration. The extracellular signal–related kinase (ERK) and mammalian target of rapamycin (mTOR) signaling pathways are required for the regulation of mGluR-LTD [29, 30] Studies have shown that antagonizing the mGluR pathway can alleviate the phenotypes of FXS [31] Therefore, mGluR theory provides new avenues for the understanding of pathological mechanisms and therapeutic intervention of FXS.

Interestingly, our findings in RNA microarray analysis revealed *GABRB3* and *BEX1* as the most significantly down-regulated and up-regulated genes*, respectively* (Supplementary Fig. S2). Altered expression of mRNA and protein for GABA receptors has been reported in *FMR1* knockout mice, implying that the loss of FMRP can affect GABA receptor subunit expression. Recent publications have also identified that the absence of FMRP can cause the upregulation of mGluR signaling, resulting in the decreased expression of *GABRB3* protein, consistent with our RNA microarray results [32–34] Therefore, the overexpression of truncated FMRP protein has profound effects on FMRP-mGluR-GABRB3 signaling pathway. *BEX1* gene may also participate in mGluR-LTD and mGluR-LTP signaling pathways. *BEX1* gene is linked to neurotrophin signaling as an interactor of the Trk tyrosine kinases (TrkA, TrkB and TrkC) or p75 neurotrophin receptor (p75NTR), regulating differentiation, growth, and survival of neuronal and glial cells [35] Trk receptors can be activated by several canonical pathways, including the phosphatidylinositol-3 kinase (PI3K)/AKT/mTOR and Ras/MAP kinase signaling pathways [36] Additionally, TrkB can participate in the LTP signaling pathways and mediate the synaptic plasticity [37] However, the signaling mechanisms of p75NTR are still poorly understood. p75NTR can change the functions of the amygdale [38] and contribute to multiple cell responses processes, such as apoptosis, survival, axonal growth, and cell death. When *BEX1* protein is overexpressed, it can inhibit the NF-κB activity by Trk receptors and p75NTR, without affecting the activation of AKT and Erk1/2 signaling [35] which are critical molecules involved in mGluR-LTD signaling pathways. The high *BEX1* level in connection with the mGluR-LTP or mGluR-LTD signaling provides new insights into the interactions of FMRP in FXS-related signaling pathways.

## Conclusion

In conclusion, our study identified a new exon from amid intron 9 of human *FMR1* gene with wide expression in normal healthy individuals. In particular, sequences similar to the new exon can be only found in genomes of primates, and its insertion can produce a truncated FMRP with altered cellular localization. Our preliminary data from RNA microarray analysis points to the possibility that *BEX1* gene may be a new player in the FXS-related mGluR-LTP or mGluR-LTD signaling pathways. However, the complicated molecular mechanisms of this new alternative exon-influenced roles of FMRP await further clarification.

## Methods

### RNA isolation and cDNA synthesis materials

Peripheral blood samples were obtained from the Department of Laboratory Medicine of 900th Hospital of the Joint Logistics Force. RNA was extracted from the peripheral blood of 52 non-FXS individuals using RNA isolation kit (Qiagen, Hilden, Germany), following the manufacturer’s protocol and stored at − 80 °C for subsequent use. The quality of RNA was evaluated by Biophotometer (Eppendorf, Hamburg, Germany). Synthesis of the cDNA was carried out following the instructions of the manufacturer (Toyobo, Osaka, Japan).

### Real-time reverse transcriptase PCR

qRT-PCR reactions were performed using iTaq SYBR Green Kits followed the 3-step cycles from the manufacturer’s protocol (Toyobo, Osaka, Japan). All reactions were carried out in triplicates and the cycles were run on Bio-Rad CFX96 real-time system (Bio-Rad, Hercules, CA, USA). The primers used were listed in Supplementary Tab. S1.

### Bioinformatics

Four softwares were used to evaluate the splicing signals in this study, including ASD (http://www.ebi.ac.uk/asd), HSF (http://www.umd.be/HSF/), BDGP (http://www.fruitfly.org/seq_tools/splice.html), and ASPicDB (http://t.caspur.it/ASPicDB/index.php).

### Western blot

We used 10% polyacrylamide gel to separate target proteins, which were extracted from cell lysates using a lysis buffer (50 mM Tris-HCl, pH 7.4, 150 mM NaCl, 1% Triton X-100, 1% sodium deoxycholate, 0.1% SDS and protease inhibitor mixture). The protein concentration was determined by bicinchoninic acid assay protein assay (Bio-Rad, Hercules, CA, USA). The proteins were transblotted onto polyvinylidene fluoride membrane (BioRad, Hercules, CA, USA), and the membrane was blocked with Tris-buffered saline (TBS) containing 5% non-fat milk for 1 h, and incubated with a mouse monoclonal antibody anti-FMRP (Abcam, Cambridge, MA, USA) at 1:750 dilution for overnight incubation at 4 °C. On the next day, the membrane was incubated with a horseradish peroxidase (HRP)-conjugated secondary antibody (Santa Cruz, Dallas, Texas, USA) at 1:5000 dilution for 1 h. The membrane was washed thrice, and enhanced chemiluminescence solution (ECL) (Beyotime, Shanghai, China) was added while exposing the film in accordance with conventional procedures. For the detection of *BEX1* protein, a rabbit monoclonal anti-BEX1 (Abcam, Cambridge, MA, USA) primary antibody at 1:1000 and HRP-conjugated anti-rabbit secondary antibody were used.

### Plasmids construction

HEK293T cells (Catalog Number: GNHu17) were obtained from Shanghai Cell Bank, Chinese Academy of Science. To identify the subcellular distribution of the end product encoded by the novel transcript containing the 140 bp sequence, we inserted the full-length coding sequence of *FMR1* or fragment containing exons 1–9 and the 140 bp sequence into the eukaryotic expression vector pEGFP-N2 (BD Biosciences, San Jose, CA, USA). Full-length coding sequence of *FMR1* gene was amplified with primers wFMR1-F and wFMR1-R (wFMR1-F: 5′-AAAGAGCTCGATGGAGGAGCTGGTGGTGGA AG-3′; wFMR1-R:5′-ACGCGCGACCGGGTACTCCATTCACGAGTG-3′;), whereas the fragment containing exons 1–9 and the 140 bp sequence was amplified with primers tFMR1-F and tFMR1-R (tFMR1-F: 5′-AAAGAGCTCGATGGAGGAGCT GGTGGTGGAAG-3′; tFMR1-R:5′-GCGTCGACCGACTTCAACCCTACTAAGT TCCTTGGA-3′). All primers contained the restrictive enzyme sites *Sac* Iand *Sal* I, for convenience of subcloning. For construction of the lentiviral overexpression vector, the target coding fragment was amplified using specific primers tFMR1-PF and tFMR1-PR with *Not* Iand *Mlu* Isites, respectively (tFMR1-PF: 5′-AAAT ATGCGGCCGCATGGAGGAGCTGGTGGTGGAA-3′; tFMR1-PR: 5′-GCGACGC GTTCAGATCTTCAACCCTACTA A-3′). After the amplified product was linked to the lentiviral vector pLEX-MCS, the packaging plasmids pMD2.G and psPAX2 (Open Biosystems, Huntsville, USA) were used to co-transfect HEK293T cells using polyethylenimine (PEI) reagent (Sigma, St. Louis, MO, USA).

### Cell culture and transfection

HEK293T cells (Catalog Number: GNHu17) were cultured in Dulbecco’s Modified Eagle Medium (DMEM) medium with 10% fetal bovine serum (FBS) and 1% penicillin/streptomycin (Invitrogen, Carlsbad, CA, USA). HEK293T cells were transfected with the recombinant eukaryotic vectors using Lipofectamine 2000 (Invitrogen, Carlsbad, CA, USA) following the manufacturer’s instructions. Three days later, the cells were observed, and RNA/protein was extracted for subsequent use. As for the recombinant lentiviral vector, transfection of HEK293T cells followed the recommended protocol (Sigma, St. Louis, MO, USA) by use of PEI reagent. The virus supernatant of cells was collected per 24 h for 3 days and used to infect a new batch of HEK293T cells. The newly infected HEK293T cells were cultured in DMEM/F12 (1,1) medium with puromycin for about 15 days. Finally, stably transfected HEK293T cells were harvested.

### Immunofluorescence

The subcellular distribution of proteins was examined by immunofluorescence staining. Specifically, the cells were fixed with 4% paraformaldehyde for 30 min, permeabilized with 0.5% Triton X-100 for 20 min, and incubated with 3% bovine serum albumin (BSA) for 1 h. Then, the cells were stained with the primary antibodies of interest, such as anti-FMRP and anti-BEX1 (Abcam, Cambridge, MA, USA) overnight at 4 °C. On the next day, the cells were washed thrice with phosphate-buffered saline (PBS) and incubated with secondary antibodies Alexa Fluor® 594-conjugated goat anti-mouse IgG or goat anti-rabbit IgG for 2 h at room temperature (Santa Cruz, Dallas, Texas, USA). The cell nuclei were stained with 4′,6-diamidino-2-phenylindole (DAPI) dye for 5 min (Beyotime, Shanghai, China). Finally, we observed cells under an FV1000 laser-scanning confocal microscope (Olympus, Tokyo, Japan).

### RNA microarray analysis

Total RNA was extracted from two batches of stably transfected HEK293T cells, including HEK293T cells transfected by void lentiviral vector (pLEX-MCS) and those by pLEX-MCS-tFMR1 vector. After the evaluation of RNA quality according to manufacturer’s protocol, RNA microarray hybridization was performed at Capital Bio Company (Beijing, China) and Affymetrix Human Genome U133 Plus 2.0 was used for analysis (Affymetrix, Santa Clara, CA, USA). For the functional analysis of the differentially expressed genes, DAVID (http://david.abcc.ncifcrf.gv/) database was also used.

### Statistical analysis

All experimental data were expressed as means ± SEM. Data were analyzed using *t-*test by using SPSS 18.0. Differences were considered statistically significant at *p* < 0.05 in all cases.

#### Accession numbers

Insertion sequence of *FMR1* gene is available from GenBank MF593118. Sequenced reads have been deposited in the NCBI Gene Expression Omnibus (GEO) database (accession number GSE101830).

## Supplementary information

**Additional file 1: Figure S1.** Comparative genomics showed that sequences homologous to the 140 bp sequence are only found in the genomes of primates. Multiple sequences alignment of FMR1 in mammals. The part underlined in green represents the140 bp novel alternative splice exon and its splicing signals, and the red square indicates the 140 bp novel alternative splice exon. **Figure S2.** RNA microarray analysis of overexpressed truncated FMRP protein and ontological classification of differentially expressed genes. **Table S1.** Sequence of qRT-PCR primers. **Table S2.** Top 20 up-regulated expression genes in HEK293T cells with overexpressed tFMRP protein. **Table S3.** Top 20 down-regulated expression genes in HEK293T cells with overexpressed tFMRP protein.

## Data Availability

The datasets used and/or analyzed during the current study are available from the corresponding author on reasonable request.
