# Supplementary material for: Further identification of a 140bp sequence from amid intron 9 of human FMR1 gene as a new exon
Source: BMC Genet. 2020 Jun 18;21:63. doi: 10.1186/s12863-020-00870-2 (PMC7301526; doi:10.1186/s12863-020-00870-2)
Supplement: Supplementary file 1 — Additional file 1: Figure S1. Comparative genomics showed that sequences homologous to the 140 bp sequence are only found in the genomes of primates. Multiple sequences alignment of FMR1 in mammals. The part underlined in green represents the140 bp novel alternative splice exon and its splicing signals, and the red square indicates the 140 bp novel alternative splice exon. Figure S2. RNA microarray analysis of overexpressed truncated FMRP protein and ontological classification of differentially expressed genes. Table S1. Sequence of qRT-PCR primers. Table S2. Top 20 up-regulated expression genes in HEK293T cells with overexpressed tFMRP protein. Table S3. Top 20 down-regulated expression genes in HEK293T cells with overexpressed tFMRP protein. [file 12863_2020_870_MOESM1_ESM.doc]

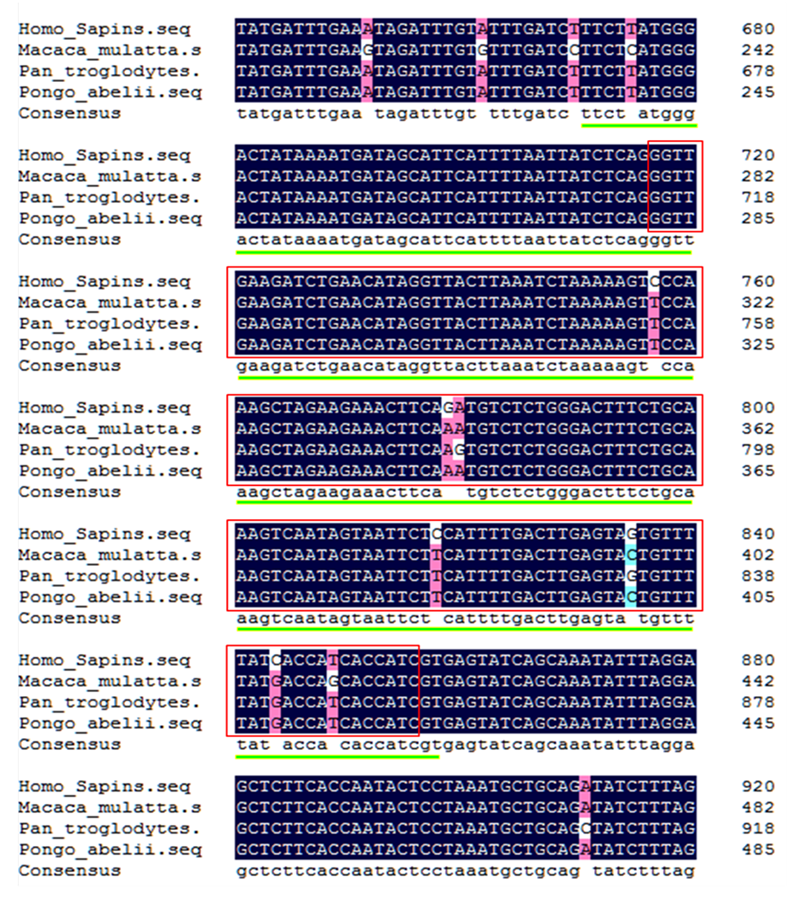


**Supplementary Fig S1.** Comparative genomics showed that sequences homologous to the 140 bp sequence are only found in the genomes of primates. Multiple sequences alignment of FMR1 in mammals. The part underlined in green represents the140 bp novel alternative splice exon and its splicing signals, and the red square indicates the 140 bp novel alternative splice exon.


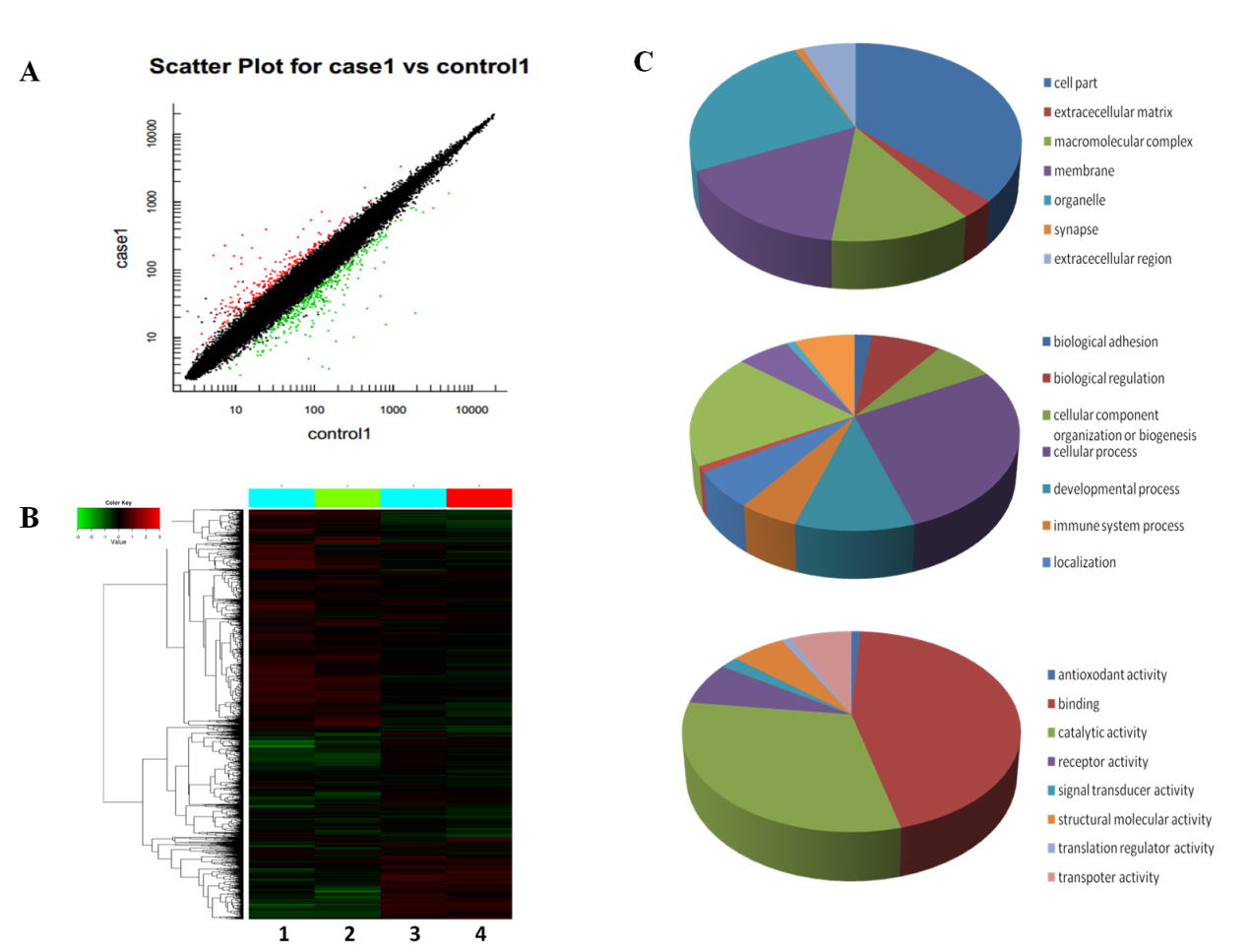


**Supplementary Fig S2.** RNA microarray analysis of overexpressed truncated FMRP protein and ontological classification of differentially expressed genes.

**(A)** Scatter plot for case 1 (HEK293T cells transfected by pLEX-MCS- tFMR1) vs control 1 (HEK293T cells transfected by pLEX-MCS). The red represents up-regulated genes, the green represents down-regulated genes, and the black represents no differentially expressed genes.

**(B)** the cluster dendrogram of RNA expression profile chip. Numbers 1 and 2: RNA analysis of HEK293T cells transfected by pLEX-MCS- tFMR1; numbers 3 and 4: RNA analysis of HEK293T cells transfected by pLEX-MCS. The red represents up-regulated genes, the green represents down-regulated genes, and the black represents no differential expressed genes.

**(C)** Ontological classification of the differentially expressed genes. 1: biological process, mainly including metabolic process (28%) and cellular process (19%); 2: cellular component, mainly including cell part (37%), organelle (25%) and membrane (16%); 3: molecular function, mainly including binding (45%) and catalytic activity (31%).

**Supplementary Table S1. Sequence of qRT-PCR primers.**

| Gene | Primer | Sequence（5’-3’） |
| --- | --- | --- |
| GABRRB3 | Forward  Reverse | TTCGCCACAGGTGCCTAT  TGATCCCGAGGGCAACTCTA |
| NAP1L2 | Forward  Reverse | TAGGCGTGTACGGTCCTTTG  ACTTCCCATCGTCTCCAAGC |
| NAP1L3 | Forward  Reverse | AACAGAGGCTAAGGCAAGGG  GTTGTGCGACTCTGGGTAGT |
| NR2E1 | Forward  Reverse | ACTGGGTTTCCCTTTAGGCTC  ATCTAAAATGCGGCCTCCTG |
| RGS-7 | Forward  Reverse | AGAAGATCCAGCCGTTTACCTC  GCTTGTGCTTCTGCTTGCAT |
| BEX1 | Forward  Reverse | TCGTCACTCGTGTCTCGCTA  GACTCCATTACTCCTGGGCCT |
| MAGE | Forward  Reverse | GTCGGCAGTCAAGCCATCAT  CGCTTGGGCTCTTAGTGGAT |
| MAGEB2 | Forward  Reverse | TCCTGACTTCCGCTTTGGAG  ATGATGGCTTGACTGCCGAC |
| PNPLA4 | Forward  Reverse | ATGGCGTTGAGTGACGGC  TTCTAGCTGTAGCACAATGCAGC |
| PPP1R1A | Forward  Reverse | GAGCCAACTCGGTCTGAGAGA  TGTTTCTTCCTTCCCAGAGGC |
| RHOB | Forward  Reverse | CCAAGCCTACGACTACCTCG  AGTTGATGCAGCCGTTCTG |
| FMR1 | Forward | GCGCTCTCTGCAATCAGTGT |
|  | Reverse | TTTGCAGAGCACGGCTGAAT |
| GAPDH | Forward | GAAGCTTACTGGAATGGCTTTCC |
|  | Reverse | GATATCATCATACTTGGCTGGTTTCTC |

**Supplementary Table S2. Top 20 up-regulated expression genes in HEK293T cells with overexpressed tFMRP protein.**

| Gene Symbol | Gene Title | RefSeq Transcript ID | Ratio |
| --- | --- | --- | --- |
| BEX1 | brain expressed, X-linked 1 | NM_018476 | 84.0336 |
| TOX3 | TOX high mobility group box family member 3 | NM_001080430 | 43.8596 |
| MAGE | melanoma antigen family B, 2 | NM_002364 | 39.6825 |
| PNPLA4 | patatin-like phospholipase domain containing 4 | NM_001142389 | 30.3952 |
| MAGEH1 | "melanoma antigen family H, 1" | NM_014061 | 21.5054 |
| ZNF595 | zinc finger protein 595 | NM_001286052 | 17.0648 |
| PLP2 | proteolipid protein 2 (colonic epithelium-enriched) | NM_002668 | 16.5289 |
| PSMD5 | proteasome (prosome, macropain) 26S subunit, non-ATPase, 5 | NM_001270427 | 12.1065 |
| DPYSL3 | dihydropyrimidinase-like 3 | NM_001197294 | 9.3023 |
| ACSS3 | acyl-CoA synthetase short-chain family member 3 | NM_024560 | 8.3542 |
| KLK8 | kallikrein-related peptidase 8 | NM_001281431 | 7.5586 |
| KLK7 | kallikrein-related peptidase 7 | NM_001207053 | 7.3692 |
| RBFOX3 | "RNA binding protein, fox-1 homolog (C. elegans) 3" | NM_001025448 | 6.4767 |
| ERBB4 | v-erb-b2 avian erythroblastic leukemia viral oncogene homolog 4 | NM_001042599 | 5.8411 |
| PTPRD | "protein tyrosine phosphatase, receptor type, D" | NM_001040712 | 5.4437 |
| SFRP1 | secreted frizzled-related protein 1 | NM_003012 | 5.2549 |
| RAB38 | "RAB38, member RAS oncogene family" | NM_022337 | 5.1020 |
| CCL2 | chemokine (C-C motif) ligand 2 | NM_002982 | 4.8780 |
| APOBEC3G | "apolipoprotein B mRNA editing enzyme, catalytic polypeptide-like 3G" | NM_021822 | 4.6147 |
| GNAS-AS1 | GNAS antisense RNA 1 | NR_002785 | 4.5351 |

**Supplementary Table S3. Top 20 down-regulated expression genes in HEK293T cells with overexpressed tFMRP protein.**

| Gene Symbol | Gene Title | RefSeq Transcript ID | Ratio |
| --- | --- | --- | --- |
| GABRB3 | gamma-aminobutyric acid (GABA) A receptor, beta 3 | NM_000814 | 0.0296 |
| DSCR8 | down syndrome critical region gene 8 | NM_032589 | 0.0324 |
| NAP1L3 | nucleosome assembly protein 1-like 3 | NM_004538 | 0.0382 |
| SV2A | synaptic vesicle glycoprotein 2A | NM_001278719 | 0.0619 |
| LRFN5 | leucine rich repeat and fibronectin type III domain containing 5 | NM_152447 | 0.0645 |
| NAP1L2 | nucleosome assembly protein 1-like 2 | NM_000814 | 0.0752 |
| PRRX1 | paired related homeobox 1 | NM_021963 | 0.0997 |
| ESRP2 | epithelial splicing regulatory protein 2 | NM_006902 | 0.1265 |
| SLC2A3 | "solute carrier family 2 (facilitated glucose transporter), member 3" | NM_024939 | 0.1303 |
| KRTAP19-1 | keratin associated protein 19-1 | NM_006931 | 0.1332 |
| NR2E1 | "nuclear receptor subfamily 2, group E, member 1" | NM_181607 | 0.1389 |
| CTSZ | cathepsin Z | NM_001286102 | 7.0024 |
| NPY1R | neuropeptide Y receptor Y1 | NM_001336 | 0.1428 |
| BEX5 | "brain expressed, X-linked 5" | NM_000909 | 0.1665 |
| TCEAL3 | transcription elongation factor A (SII)-like 3 | NM_001012978 | 0.1696 |
| FAM26F | "family with sequence similarity 26, member F" | NM_001006933 | 0.1832 |
| NELL2 | NEL-like 2 (chicken) | NM_001010919 | 0.1875 |
| C8orf4 | chromosome 8 open reading frame 4 | NM_001145107 | 0.1943 |
| N4BP2L1 | NEDD4 binding protein 2-like 1 | NM_020130 | 0.2094 |
| RGS7 | regulator of G-protein signaling 7 | NM_001282773 | 0.2115 |
